# The Human Wallace Line: Racial Science and Political Afterlife

**DOI:** 10.1017/mdh.2019.29

**Published:** 2019-06-18

**Authors:** Fenneke Sysling

**Affiliations:** Department of History and Art History, University of Utrecht, Drift 6, 3512 BS Utrecht, The Netherlands

**Keywords:** Anthropometry, race, Southeast Asia, Alfred Russel Wallace, human diversity

## Abstract

This paper examines racial science and its political uses in Southeast Asia. It follows several anthropologists who travelled to east Nusa Tenggara (the Timor Archipelago, including the islands of Timor, Flores and Sumba), where Alfred Russel Wallace had drawn a dividing line between the races of the east and the west of the archipelago. These medically trained anthropologists aimed to find out if the Wallace Line could be more precisely defined with measurements of the human body. The paper shows how anthropologists failed to find definite markers to quantify the difference between Malay and Papuan/Melanesian. This, however, did not diminish the conceptual power of the Wallace Line, as the idea of a boundary between Malays and Papuans was taken up in the political arena during the West New Guinea dispute and was employed as a political tool by all parties involved. It shows how colonial and racial concepts can be appropriated by local actors and dismissed or emphasised depending on political perspectives.

## 1 Introduction

The human diversity of insular Southeast Asia has long been a source of wonder and the subject of various strategies of scientific inquiry. In the course of the nineteenth and twentieth centuries, insular Southeast Asia attracted natural historians, medical experts, physical anthropologists and (more recently) geneticists, each hoping to explain the supposed variety in human types. They were, in a region that until 1945 (New Guinea 1962) was for the most part under the control of the Dutch Empire, an international, mostly European, group of scientists, including – among others – French craniologists, British and German naturalist travellers and Dutch anthropologists.

One important structuring concept for the human diversity in this region was the difference between people with frizzy hair and darker skins in the east and light brown people with straight hair on the western islands. The difference between these two people had already been noticed by early European travellers, together with the difference in flora and fauna. After extended travels in the region, the British naturalist explorer Alfred Russel Wallace – better known as the co-discoverer with Darwin of evolution by natural selection – proposed in the 1860s that the region could be divided into two biological zones, separated by what came to be known as Wallace’s line.^1^ As well as this famous faunal line, separating the woodpeckers and orangutans from the cockatoos and wallabies, Wallace defined a human line, separating Malays in the west from Papuans in the east.^2^

This paper analyses the anthropological Wallace Line and its political afterlife. It starts with the work of the early naturalist travellers who conceptualised the line and continues with the engagement of a generation of physical anthropologists with the line in the late nineteenth and early twentieth centuries. Educated in medical schools, these physical anthropologists considered themselves experts in the human body, and this included the body in racial terms. Physical anthropology as a discipline had crystallised within medical departments in Europe, where encounters with unfamiliar bodies had led medical scholars to rethink both medical theories and the nature of human diversity. For those medically trained anthropologists who travelled overseas, racial differences and evolutionary hierarchies were an essential part of how they understood individual bodies and society.^3^

From the 1880s onwards, physical anthropologists were eager to measure the physical differences between Malays and Papuans and hoped to provide a line that was drawn even sharper and more objectively. Several anthropologists in this period travelled to the Timor archipelago or east Nusa Tenggara – the eastern part of the Lesser Sunda Islands, including the islands of Flores and Timor and the adjacent smaller islands, crossed by the anthropological Wallace Line – and did fieldwork among the Atoni, Manggarai or Savunese. This paper shows that even though this generation of anthropologists failed to substantiate their claims, the idea of a boundary between Malays and Papuans persisted. The human Wallace Line became salient, especially during the West New Guinea dispute, and was employed as a political tool by all parties involved to further their goals.

Exploring the making and afterlife of the Wallace Line, this paper follows historians of medicine, race and geography who have shown how the idea of a boundary line was an important organising principle in the making of (racial) conceptions of the word and how it shaped realities.^4^ Not only for map-makers and geographers but also for biologists, ethnologists and medical experts, maps were a visual tool to abstract knowledge and to circulate it. In her article about Wallace’s use of maps, Jane Camerini argues that for Wallace ‘maps of faunal regions served as instruments both of thought and of persuasion’.^5^ In racial science, maps simplified diversity and emphasised racial difference. As Veronika Lipphardt writes: ‘To visualise their view of human evolution and the geographical distribution of races, scientists painted arrows, drew lines, or hatched zones onto geographical maps of the world, or Europe, or any other continent’, even though the data they collected did not usually allow for drawing clear-cut boundaries.^6^

Wallace’s maps, both the faunal and the human, were important scientific tools, but the reproduction of the Wallace Line consisted of more than maps. The idea of a human line was mobilised in academic texts and in the speeches of politicians, so it was the spatial imagery in a broader sense, rather than the cartography, that proved to be influential.^7^ Work by historians of colonial medicine on these spatial imageries has been pivotal in drawing our attention to the making of colonial hierarchies. They have shown how inequalities in healthcare resulted from and led to colonial oppression and racial prejudices. The construction of boundaries was often central to the work of colonial doctors: between the sick and the healthy, the clean and the dirty, and in these categories health and race were messy, entangled categories. These boundaries could also have real consequences on the ground, as in the case of *cordons sanitaires*.^8^

This paper follows the most recent scholarship in colonial science and medicine that explores how colonial subjects ignored or resisted Western medicine, how they integrated it with their own health practices and, similarly, how they created alternative versions of racial ideas.^9^ This paper shows how the idea of a boundary line could be reactivated and appropriated by indigenous actors. In one of the few examples of a similar development, Stephanie Lawson traces the history of the Melanesia/Polynesia divide and shows how the idea of Melanesia was once developed in racialist ethnography but was appropriated and changed into a term of empowerment by Melanesians.^10^ While this article emphasises the racial history of Wallace’s division and the way it was rooted in colonial ideas, its continued relevance among Indonesians and Papuans is also taken seriously.

## 2 East and West

The idea of a contrast between the eastern and western peoples of the region and of a boundary line or region between them was first conceived by the generations of naturalist travellers that preceded Wallace. Father and son Johann Reinhold and Georg Forster, for example, naturalists who travelled with James Cook on his second voyage to the Pacific, observed ‘two great varieties’, one ‘more fair, well limbed athletic, of a fine size and a kind benevolent temper; the other blacker, the hair just beginning to become woolly and crisp, the body more slender and low, and their temper, if possible more brisk, though somewhat mistrustful’.^11^ The French Jules Dumont d’Urville contrasted the black colour of Papuans with that of their ‘copper-coloured’ neighbours.^12^ In the nineteenth century, John Crawfurd and George Windsor Earl confirmed this division. Their claim to reliability was a longer stay in the region, even though Crawfurd, who knew the island of Java well, never travelled to New Guinea. Crawfurd, the British resident of Yogyakarta during the British interregnum (1811–16), wrote that the region knew two aboriginal races, a straight haired ‘brown’ and a woolly-haired ‘negro’ race, the latter being the inferior one.^13^ George Windsor Earl, on the other hand, in his book on the Papuans, reiterated the idea of two races and called the Papuans less civilised though ‘physically superior to the races of Southeast Asia’.^14^

**Figure 1:**
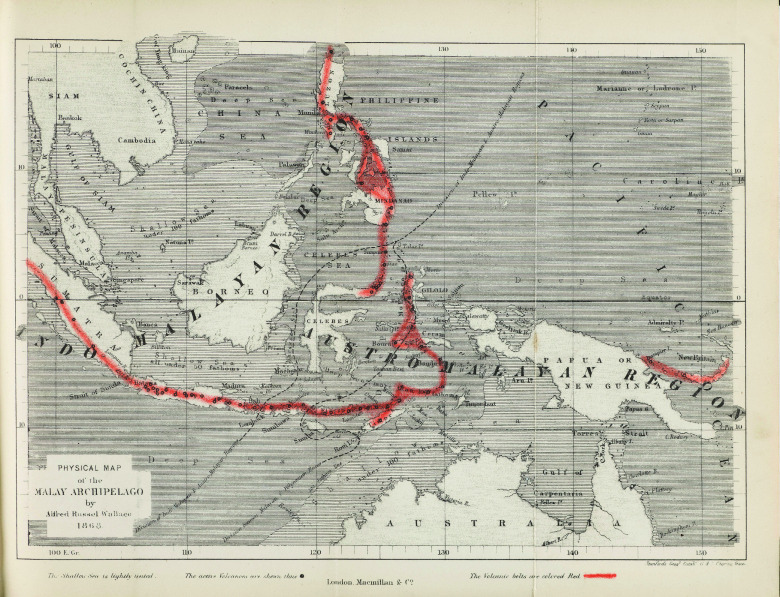
Map from Wallace’s *The Malay Archipelago* (1869). The dashed line on the left is the original, zoological, Wallace Line; the one on the right is the racial dividing line. Wallace also highlighted the region’s volcanic belt. Source: Wellcome Library, London.

Wallace travelled in the Indonesian archipelago from 1854 to 1862, observing wildlife and indigenous people at every stop he made. This journey led him to the concept of what came to be known as ‘Wallace’s line’, the faunal dividing line between Australia and Asia.^15^ Wallace also carried on the work of Crawfurd and Windsor Earl and conceptualised a boundary line for humans. This human line between Malays and Papuans was placed east of the line for fauna: it separated the islands of Sumba, Flores and Timor from Sumbawa. Wallace’s book *The Malay Archipelago* (1869), in which he recounts his travels and biogeographical theory, includes a map with both lines (see Figure 1).^16^

Wallace’s anthropological line has only recently received due attention from historians, first of all from Jeremy Vetter. As Vetter has shown in his analysis of the line, Wallace’s ‘human line’ was based on direct observations and trained judgement. When he landed on New Guinea, Wallace identified immediately that Papuans were essentially different from Malays: ‘The loud rapid eager tones, the incessant motion, the intense vital activity manifested in their speech and actions, are totally the reverse of the quiet unimpulsive unanimated Malay.’^17^ Both Vetter and Chris Ballard show that Wallace, like Crawfurd and Windsor Earl, based his theories not only on physical features but also on mental and moral assumptions.^18^ In the colonial context of the Dutch Empire, says Vetter, ideas of Papuans as inferior fit comfortably with the presence of Papuan slaves.^19^ Wallace did attempt to compare his personal observations with the quantitative data of skull measurements of Pacific people in British craniologist Joseph Barnard Davis’s *Thesaurus Craniorum*. He concluded that the small sample size made it impossible to extrapolate and that within-group variation was greater than between-group variation.^20^

Wallace’s anthropological line was not immediately accepted by fellow scholars. Dutch geographer Pieter Robidé van der Aa disagreed with the sharp distinction drawn by Wallace. Besides giving linguistic and ethnological evidence, he argued that while Papuans were usually defined by their black skin and their hair, not all Papuans in the Indonesian archipelago were particularly black, nor was their hair growth always in tufts. Robidé proposed that there was one race in the archipelago, the ‘insular race’.^21^ Others mentioned the exceptions to the line. The German geographer Carl Meinicke criticised Wallace because the inhabitants of the Moluccas were not a mix of Papuans and Malays but included an aboriginal group.^22^ Pieter Veth, Wallace’s translator and propagator in the Netherlands, agreed with Meinicke, and also emphasised the presence of Malays on the Moluccas and on Timor, far east of the line.^23^ In France, anthropologists Armand de Quatrefages and Ernest Hamy, who based their ideas on their skull collection and had never been to Southeast Asia, agreed that Wallace had simplified the complexity of the islands. One of the things that Wallace ignored, they said, was the presence of so-called Negritos, groups of people of small stature who were found on each side of Wallace’s anthropological line.^24^

One focus of the discussions was the boundary region, and especially the island of Timor, which on Wallace’s map was just east of the line, on the Papuan side. Wallace’s line was a simplified generalisation of his observations, and he suggested that there was a boundary region where characteristics from each type blended. Crawfurd too had suggested the existence of a transitional type existing on Flores, Timor and the Moluccas.^25^ However, travellers in this period were far from being in agreement about the inhabitants of Timor and the nearby islands. Salomon Müller, a German naturalist traveller, wrote in 1857 that, contrary to the rumours, he had seen no Papuans [*Austraal-negers*] living in the mountains of Timor, only the straight-haired and yellow Malays. The Frenchman Pierre-Adolphe Lesson, on the other hand, wrote that the inhabitants of Timor were closer to the black race than to any other.^26^ Based on two skulls from Timor in their craniological collections, which Hamy and Quatrefages thought clearly combined Malay and Papuan features, and their knowledge of other collections, Hamy argued that Papuan influence on Timor was a fact, despite its mixed population that also included a Negrito ‘element’.^27^

Quatrefages and Hamy did take up the idea of a boundary region: ‘The geographical distribution of human races,’ they concluded, ‘like that of animals and plants, leads us to consider the region where Timor is located as a boundary region, having both Asia and Oceania as its natural history, that is: Timor is () the most western [island] where Papuans have settled.’^28^ That the main division of races in the archipelago was the one between Malays and Papuans now became established in writings about the Indies, including the idea that there was a boundary region.^29^ But where exactly? The racial make-up of the boundary zone, crossing the Moluccas and the Timor archipelago with its different islands, was still in question, and it was to these islands that following generations of anthropologist would travel with their measuring instruments.

## 3 Anthropologists and the Wallace Line

Anthropometry, the study of measurements of the living human body, was one of the subdisciplines of racial science in the nineteenth and twentieth centuries.^30^ It was with the strengthening of European power in the non-European world in the second half of the nineteenth century that racial science developed, including the ideas that race was an essential attribute of people and that scientific methods were able to distinguish between races and types. In addition, evolutionary theory provided a framework to naturalise racial hierarchies. In Southeast Asia, Malays were often classified as more advanced: after all, they had been able to build the Borobudur and Prambanan temples on Java. Papuans and Negritos, on the other hand, were put lower on the ladder of civilisation and their lives compared to those of Europeans in the Stone Age.

Physical anthropologists almost always had a medical education; they combined craniology, the study of skulls and skeletons, with the study of living people to understand the distribution and evolution of the races of mankind. Skulls and bones of non-European people had arrived in Europe since the earliest explorations and were seen as parts of the body that could not be changed by the environment: that is, a place where racial difference could be found. However, anthropologists in the late nineteenth century increasingly found the living human body worth measuring, especially since there were always more living individuals than there were skulls in skull collections. ‘The small number of skeletons which our museums contain,’ wrote the French anthropologist Paul Topinard in 1881, ‘is absolutely insufficient, and living people alone can supply the number indispensable to arrive at any degree of certainty.’^31^ For anthropometrists, the view that quantified data of as many human bodies as possible was best became a professional desideratum, and they distanced themselves from the descriptive methods of earlier naturalist travellers, even as they too relied on their impressions more than they admitted.^32^ In the twentieth century, these morphological studies were supplemented with genetic research on the distribution of blood groups and the ability to taste the bitterness of phenylthiocarbamide (PTC).^33^

Anthropometry, then, was both a reaction to and an extension of osteological research, but it had its own disadvantages, such as disagreements on the definitions of measuring-points. Many German-speaking anthropologists in the twentieth century followed the methods of Rudolf Martin, whose measuring-points were, according to a reviewer, ‘designated with marvellous exactness, every single one being fixed linguistically and defined etymologically’, but they did not bring an end to the practical difficulties of measuring something as complex as the human body.^34^ Anthropometry, criticised the German anthropologist Richard Neuhauss in 1911, produced heaps of numbers that after a decade no one would understand anymore.^35^

Even when done correctly, anthropologists discussed among themselves how useful these measurements really were. While they did use quantitative data to map the races of mankind, their material also challenged racial classifications or turned out to be inconclusive.^36^ Therefore, direct observation and the sharpened eye of the experienced anthropologist continued to complement measurements to recognise differences between people that were not susceptible to explanation by numbers. If measurements and ‘trained judgement’ did not add up, friction followed, which could be overcome by emphasising one of the strategies for knowledge and diminishing the other, incompatible one. Anthropologists were also happy to defer conclusions to future anthropologists and to admit that local circumstances only complicated easy categorisations.^37^ This tension concerning the question of what exactly numbers could say continued to define the discipline far into the twentieth century and was never fully resolved.

At the same time, the racial categories that anthropologists produced were not always the kind of knowledge colonial administrators considered suitable for governing purposes. Recent studies on the relation between empire and the human sciences, such those by Helen Tilley and Erik Linstrum, have argued that even though these sciences aimed to produce knowable and governable subjects, they also questioned and undermined colonial categories and values.^38^ Similarly, as we will see below, anthropologists were frustrated in their attempts to find a human Wallace Line. At the same time, however, this racial boundary line outlived these frustrations, not among colonial administrators but among colonial subjects.

Some of the first anthropometric measurements in the Indonesian archipelago were taken by Karl Scherzer and Eduard Schwarz, doctors and anthropologists of the Austrian *Novara* expedition, which visited Java in May 1858. They took as many as eighty different measurements per person from soldiers and prisoners in Batavia,^39^ and many subsequent measurements were also taken in disciplinary institutions of the colonial state. While prisons, hospitals and schools had the advantage for the anthropologists that resisting the measurements was hardly an option, the disadvantage of these locations according to the anthropologists was that they usually held a mix of people. Anthropologists preferred research subjects of ‘pure race’ and located in ‘remote’ places, where the infrastructure of colonialism had not yet penetrated. In those places, the encounter between researchers and subjects involved a process of coercion, persuasion or negotiation. The measurement itself was an intimate affair, with the instruments being applied to different parts of the body and responses varying from fear through confusion to curiosity. Some locals may have seen the measurements as a pleasant diversion from everyday routine; for many others it was a frightening experience. Sometimes local villagers just refused to show up, which left the anthropologists complaining about the gaps in their data.

In the Dutch Indies, visiting people in ‘remote’ places was increasingly achievable in the late nineteenth century because of the growing medical infrastructure and the opening up of the outer areas of the archipelago. In a phase of new imperialism in the late nineteenth century and the early twentieth, the islands further away from the colonial centre of Java were, often violently, brought under Dutch control. This went hand in hand with the new Ethical Policy, the Dutch version of the civilising mission that emphasised bringing modernity to those who were in need of it. This meant that on many islands, especially in the hinterlands, people who had been part of the Dutch Empire only formally were now confronted with the presence of European men, from missionaries and administrators to medical experts eager to measure, observe and collect in places that were not frequented by Westerners.^40^ The latter were encouraged by the government with the familiar colonial argument that a modern colonial state should know its subjects. It was often military medical doctors stationed at the outer islands, or doctors on vaccination campaigns, with an interest in physical anthropology and eager to add something to scientific knowledge, who were presented with opportunities to study lesser known people. The Dutch colonial government also supported racial scientists by sending out a manual for natural history and anthropology to Dutch administrators in the 1890s, including a ready-to-use form for anthropometrical data.^41^

Dutch anthropologist Herman ten Kate, who visited the Timor archipelago in the 1890s, embodied this new generation of professional racial scientists who profited from the opening up of the Dutch Empire. Trained as a doctor, ten Kate studied the latest developments in the field of physical anthropology in Paris, Berlin, Göttingen and Heidelberg. He had research experience in the anatomical collections of Europe but also emphasised the importance of on-the-spot observations and measurements. He was a classic proponent of the new physical anthropology, emphasising the objectivity of numbers and preferring photography to drawing. He aimed to find out whether Wallace’s racial observations were quantifiable and hoped to be able to draw a more precise line based on these measurements. He was also a salvage anthropologist, believing that data must be collected soon, before pure races were ‘submerged’ by ongoing modernisation and evolution.^42^

In 1891, ten Kate travelled to the Dutch Indies, invited by the Royal Dutch Geographical Society to do anthropological research on the island of Flores, close to where the Wallace Line separated Flores and Sumba from Sumbawa. By the time ten Kate arrived in the Indies, the Dutch were carrying out military raids on Flores, and it seemed safer to be based on Timor. He was based in Kupang on the coast but generally avoided coastal towns because racial pureness was thought to increase with distance from the coast. From Kupang, ten Kate set out on trips into the interior and took boats to the smaller islands in the vicinity: Semau, Roti, Sawu, Sumba, Solor, Adonara and eventually Flores, bringing along his assistants and a translator.

Ten Kate took anthropometrical measurements whenever the opportunity presented itself, amounting to a total of 1,318 individuals measured. If there was not much time, cephalic index (the relation between the length and the width of the head), nasal index, height, skin colour and eye colour were essential measurements. If there was time for more extensive research, ten Kate used the dynamometer to test muscular strength, and he also tested sight, sense of colour and skin sensitivity. He concluded that the Timorese were relatively immune to physical pain.^43^ That locals – among many other feelings they must have had – considered the measurement as a performance that could be ridiculed can be glimpsed when ten Kate describes how on the island of Sumba a local man copied his measurements:

That one can find fine observers full of humour among the Sumbanese sometimes became apparent when, late in the afternoon, a man held an anthropometric séance in his own way and copied all my manipulations of that morning to another subject, with a wooden stick and a long blade of grass giving me questioning looks repeatedly.^44^

The local audience laughed out loud about this performance.

To decide where a racial boundary line could be drawn, ten Kate sought the westernmost influence of Papuan physical features. Timor, he concluded, was very mixed. He wrote that the autochthonous ‘wild’ population was generally rather small in size (men measured on average 1.597 m and women 1.496 m) and had a warm dark-coffee skin colour (nos. 29–30 on the Broca colour scale) and ‘Negroid’ foreheads. Cranial indices showed that the population was neither particularly brachycephalic nor dolichocephalic.^45^ Dolichocephaly, or long-headedness, was associated with Papuans but also with pre-Malays, another ‘aboriginal’ race of the western part of the Indies, and was often tied to lesser civilisation and less use of the brain, though all these were contested, and Timor did not bring any clarity in the case.^46^

Attentive to the extent of Papuan influence in the boundary area, ten Kate encountered what he saw as the as the purest representatives of the ‘negro race’ in the highlands of the island of Flores. He based this on his quick observation of skin colour and hair form but also on the noisy clatter and chatter of the locals, so his personal impressions here carried more weight than his measurements. His servants, likely Malay carriers, confirmed his opinion. Ten Kate wrote that his opinion was confirmed by anthropometric measurements (of six men): four of them were quite tall and several were very long-headed, so ten Kate defined that as their Papuan element.^47^ The Sumbanese, on the other hand, were classified by ten Kate as closest to the pre-Malay type as could be found in the Timor archipelago. They made a better impression on him than people from the other islands, partly because they were easily persuaded to be measured.^48^ Although the region, according to ten Kate, was very heterogeneous and he encountered many types that reminded him of all sorts of other races (Hindu, American Indian), and physical markers did not follow the map at all well, he concluded that the difference between Flores and Sumba stood out the most. Therefore, he suggested that it was between Sumba, on the one hand, and Flores and Timor, on the other, that the dividing line should be drawn.^49^

Two decades later, anthropometry in the Dutch Empire had taken off with studies all over the region. In the Timor archipelago, Hendrik Bijlmer, Doeke Brouwer and Wilhelmina Keers all took measurements in the region in the wake of ten Kate, again hoping to bring more clarity in the case of the Wallace Line but finding instead a complexity that defied easy categorisation. Bijlmer would be better known for his anthropological work on New Guinea, but he started his anthropological work as a military medical officer in the Timor archipelago. The appeal of the region had not changed: in the words of Bijlmer, ‘the so-called Malays from the Western part of the Indian Archipelago met here with the off-shoots of the Oceanic Negroes whose birth-place is commonly said to have been somewhere in Melanesia, New Guinea included’.^50^

Bijlmer measured hundreds of people at eight different places on Timor, Flores and Sumba. His wife accompanied him and was responsible for the photography. He decided not to measure ‘anything that the compasses can grasp’ but those human dimensions where he expected to find racial difference, such as stature, distance between the corners of the eyes, length and width of the head (for the cephalic index) and length and width of the nose (for the nasal index), and he focused on the face with four indices and the interorbital breadth.^51^ While ten Kate wove the descriptive part of his research into his travel narrative, Bijlmer put it in a separate chapter, describing skin colour, hair form, Mongolian eye-fold and the mouth, features he classified as harder to define objectively than the other characteristics but nonetheless important.

Bijlmer had hoped that clear influences of each dimension would be traceable in the Timor archipelago. ‘No greater difference is imaginable,’ he wrote, ‘between the brown straight-haired, slightly Mongoloid Malay and the dark, frizzy-haired, absolutely non-Mongoloid Papuan.’^52^ He found, however, that the inhabitants’ hair form and skull index didn’t fulfil his hopes. He did see differences, though: Timor and Flores, according to Bijlmer, knew Melanesian elements, too, so these islands were indeed the place where two races met, while Melanesian influence was absent on Sumba, just as ten Kate had concluded earlier. Bijlmer saw the clearest human differences between Sumba and Timor, so ‘if one wanted to distinguish in the Dutch East Indies only Malays and Papuans or Indonesians and Melanesians, one would have to place the boundary between those two races undoubtedly between Sumba and Timor’.^53^

According to Bijlmer, however, the line crossed the island of Flores, but it was difficult to know where. ‘We have been accustomed,’ Bijlmer wrote, ‘to consider the frontier between Ngada and Manggerai as the Western limit of the Melanesian element.’ So when Bijlmer crossed from the Ngada region to the neighbouring Manggarai, he was full of expectation, even though Wallace had not conceptualised a sharp line but a region in which different features blended. For Bijlmer, it turned out to be a disappointment in terms of physical features. Instead, he resorted to thinking in terms of civilisational hierarchy: ‘there is certainly a great difference between the fierce-looking unwashed and roughly clothed Ngadanese and the better groomed Manggerai, mostly in Malay dress’.^54^

On eastern Timor, according to Bijlmer, Melanesian influence was most prominent. The inhabitants of this region were ‘[m]ore uniform, more true to type’, as they showed ‘great mutual resemblance’, which suggested to anthropologists that they were of a pure kind. Unfortunately for Bijlmer, they included both long-skulled and short-skulled individuals. This meant he had to conclude that both represented a specific Melanesian type and that skull type could not be a marker for difference between Malays and Melanesians. Bijlmer decided not to differentiate between these two groups, showing that he attached greater importance to the characteristics he observed than to skull shape. Similarly, as he had already decided on the basis of his impressions that Sumba was fully (pre-) Malay, he linked the medium-length skull type he found there to a pre-Malay strain.^55^

Several other doctors and anthropologists added their measurements to those of ten Kate and Bijlmer. In the 1930s, Doeke Brouwer, a military doctor on the island of Alor, east of Flores, studied the inhabitants of Alor and the smaller nearby Pantar. Measuring twenty different dimensions and indices, he divided the population on the two islands into seven groups and classified them generally as Melanesians with mostly Papuan but also pre-Malay influence. According to his findings, then, western influences were traceable even east of the line.^56^ Wilhelmina Keers, one of the few female anthropologists, visited the Netherlands Indies in 1937 and 1938 and found a diverse amalgam of groups, who hardly seemed to mix, so features differed markedly between villages. The boundary of pre-Malay influence, according to Keers, ran across the Timor islands, and she did not trace it anywhere further east.^57^ Though the measuring techniques of anthropologists had not been able to define racial differences, the anthropological Wallace Line continued to be a guiding principle in the region.

## 4 The Politics of the Wallace Line

If anything can be concluded from the politics and non-politics of the racial Wallace Line, it is that they became active at a politically opportune moment. For the Dutch colonial government, a dividing line between Malays and Papuans did not fit the image they wanted to create. Until the independence of Indonesia, the government preferred that of a patchwork of different ethnic and racial groups under one Dutch umbrella.^58^

For the Portuguese colonisers of East Timor, on the other hand, the Wallace Line, if redrawn slightly to the east, supported Portuguese colonial claims perfectly, as Ricardo Roque has shown in a relevant recent article. Ten Kate, Bijlmer and other Dutch anthropologists found that, surprisingly, the island of Timor, the western part of Dutch Timor, seemed more Melanesian than the east, which went against the wider picture of a Papuan east versus a (pre-) Malay west. The Belu of the eastern highlands were also the main ethnic group in Portuguese East-Timor, which was considered advantageous by the Portuguese, because by defining Portuguese subjects as more Malay and less Papuan, they could be portrayed as evolutionarily more advanced. In 1916, the Portuguese anthropologist António Mendes Correia suggested that East-Timor be placed west of the Wallace Line, giving the line an extra loop around East-Timor that neatly followed the border between Dutch and Portuguese Timor.^59^

In the first half of the twentieth century, Papuans had a different experience from many other colonised people of the archipelago, as the Dutch started to invest heavily in the region only after 1949, when it was their last remaining colony in the east. Papuans also played only a small role in the independence struggle of Indonesians. However, Indonesian nationalists had included West New Guinea in their discussions about territory since the early twentieth century, when they conceptualised the future Indonesia as a continuation of the current Dutch Empire rather than a racial community.^60^ Sukarno, Indonesia’s first president, was a proponent of this view, as was Mohammad Yamin, politician and theorist of Indonesian identity. Indonesia’s first vice-president, the nationalist Mohammed Hatta, had a more pragmatic preference for an Indonesia without New Guinea, but he was overruled.^61^

Indonesia proclaimed independence in 1945, but after the war that followed and the negotiations with the Dutch, West New Guinea was not part of the transfer of sovereignty and continued to be contested terrain. The Dutch concentrated all their colonial efforts on New Guinea, with the aim of uplifting the population from their primitive state. Because of the continuing presence of the Dutch in New Guinea while the Indonesian government claimed it was part of Indonesia, relations between Indonesia and the Netherlands soured until the situation escalated in 1962. The administration of West New Guinea was then transferred to the United Nations Temporary Authority and less than a year later, in 1963, to Indonesia.

The Dutch defended their prolonged presence in New Guinea with, among other things, the argument that the Papuans were racially (and linguistically and culturally) different from Malays in Indonesia. A New Guinea Committee in 1950 was supposed to prepare for negotiations but only revealed irreconcilable differences, especially on the question of whether West New Guinea belonged to Indonesia. The final, Dutch report of the Committee argued that

to elaborate on the anthropological difference between Indonesians and Papuans is () unnecessary. They each belong to different major races of mankind [white, Asian, black]. The typical mongoloid features of Indonesians will not be found among Papuans, whether they live on the coast or in the mountains.^62^

This argument became problematic, as Vincent Kuitenbrouwer found, ‘after 1951 when UNESCO published its first report on race in which scientists argued it to be a “social myth” and not a biological phenomenon’. Dutch politicians then decided that the race argument should be avoided in future publications ‘as it could be interpreted by the international community as a form of discrimination’.^63^

Papuans also invoked the racial difference between themselves and Indonesians. They had been the subject of European racial theories describing them, with their black skin and woolly hair, as primitive or as noble savages, but now they redeployed these ideas. They tried, as David Webster has shown, ‘to turn “race” into a diplomatic asset’.^64^ Based on the New Guinea Committee report mentioned earlier, a delegation of Papuans to the Netherlands argued: ‘Indonesia asserts that Papuans are Indonesians. However, science has established indisputably that Papuans and Indonesians belong to different major races. () It is also a definite fact that we don’t feel Indonesian.’^65^

Papuan leaders also sought contact with African states, emphasising their racial similarity and their common struggle against colonialism. In one of their pamphlets, *The Voice of the Negroids of the Pacific to the Negroids throughout the World*, Papuans were described as radically different from Indonesians. ‘Our country and our people,’ the pamphlet said, ‘are threatened to be handed over to our enemy, the Indonesian Republic, that is belonging to quite another race, i.e. the Mongoloïd one to which we don’t belong.’ Humorously, they continued: ‘Soekarno [the then president of Indonesia] likes to state that Papuan [sic] are Indonesians. What keeps him from claiming that butterflies belong to the family of spiders?’^66^

Indonesians, on the other hand, made the opposite claim. Sudjarwo, Indonesia’s representative to the UN, said in 1954: ‘it has also been asserted that no racial links exist between the people of West Irian [as West Papua was often called in this period] and those of the rest of Indonesia. This is not true’. He also mocked the Dutch argument: ‘It may, in fact, be asked: what kind of racial links have the Dutch with the West Irians? Certainly, I look much more like a West Irian or Papuan than do my Dutch friends!’^67^ In doing so, Indonesia rejected, on this occasion, European racial classification. At the same time, Indonesian rhetoric continued to be suffused with ideas of the primitiveness of Papuans.^68^

Since West Papua became part of Indonesia, Papuan nationalists have continued to emphasise their difference from Indonesians. This shows the resilience of an idea that was partly constructed by racial scientists but that was made more ‘real’ and relevant in the political context in which Papuans found themselves. Indonesia’s government, on the other hand, has wielded its influence to undo the boundary, not only by repressing Papuan voices but also by actively changing the demographic character of West Papua with the migration of Indonesians from other islands to the Papuan provinces.^69^

## 5 Conclusion

This article has shown how the anthropological practice of drawing lines was a forceful tool to establish racial categories. Wallace’s line was a projection of observations onto geographic space and etched the idea of a boundary onto the minds of scholars. The imagined boundary between Malays and Papuans has been a consistent feature of our conceptions of Southeast Asia, even though the line has moved on the map over time. First drawn by Alfred Russel Wallace west of the Moluccas, Flores and Sumba, the generation of physical anthropologists moved it slightly to the east. Anthropometrists hoped to find the right markers that could be used to define the difference between Malays and Papuans quantitatively, but it turned out to be impossible to link characteristics such as the cephalic index to one of the two groups. Still, like the natural historians that preceded them and the political agitators of the 1950s and 60s, they defined people as Papuan or Malay based on their observations of physical features, perceived civilisation and behaviour. Though impossible to grasp with measuring rods and calipers, the concept of an anthropological Wallace Line was (and is) flexible enough to be invoked time and again.

In the period of decolonisation, the Timor archipelago and the Moluccas were generally seen as part of Indonesia, hence the line was moved further eastwards. The discussions surrounding the transfer of sovereignty of West New Guinea from the Dutch to the Indonesians show that the idea of the human Wallace Line was there to be called on when the argument was advantageous for one of the groups involved: ‘no one can draw a distinct dividing line between the so-called Papua and Malay areas!’, said an Indonesian pamphlet in 1956, invoking it.^70^ In general, the emphasis on the difference between Malays and Papuans has been to the detriment of the Papuans, who were, in the colonial era and afterwards, considered noble savages in the most positive sense but also as evolutionarily behind, primitive and dirty. Yet no matter how steeped this racial idea was in the history of colonialism and European racial science, Papuans turned into a more positive and powerful identity tool to strengthen their links with Africa, to call for independence and to position themselves in the world.^71^

## Footnotes

1.
Jane R. Camerini, ‘Evolution, Biogeography, and Maps: An Early History of Wallace’s Line’, *Isis*, 84 (1993), 700–27, and Bernard Michaux, ‘Alfred Russel Wallace. Biogeographer’, in Charles H. Smith and George Beccaloni (eds), *Natural Selection and Beyond: The Intellectual Legacy of Alfred Russel Wallace* (Oxford: Oxford University Press, 2008), 166–85. In biographies of Wallace, the Wallace Line usually receives only brief mention compared to Wallace’s co-discovery of evolution. See, for example, Martin Fichman, *An Elusive Victorian: The Evolution of Alfred Russel Wallace* (Chicago: University of Chicago Press, 2004), 50–1, and John van Wyhe, *Dispelling the Darkness: Voyage in the Malay Archipelago and the Discovery of Evolution by Wallace and Darwin* (Hackensack, NJ: World Scientific, 2013), 149–52.

2.
For a history of the term ‘Malay’, see Anthony Reid, ‘Understanding Melayu (Malay) as a Source of Diverse Modern Identities’, *Journal of Southeast Asian Studies*, 32 (2001), 295–313; for a history of the term ‘Papua’, see J.H.F. Sollewijn Gelpke, ‘On the Origin of the Name Papua’, *Journal of the Humanities and Social Sciences of Southeast Asia*, 149 (1993), 318–32.10.1017/s002246340100015719192500

3.
Warwick Anderson, *The Cultivation of Whiteness: Science, Health, and Racial Destiny in Australia* (Durham, NC: Duke University Press, 2006); Alexandra Widmer and Veronika Lipphardt (eds), *Health and Difference: Rendering Human Variation in Colonial Engagements* (New York: Berghahn Books, 2016).

4.
Brian J. Harley, ‘Maps, knowledge and power’ in Denis Cosgrove and Stephen Daniels (eds), *The Iconography of Landscape: Essays on the Symbolic Representation, Design and Use of Past Environments* (Cambridge: Cambridge University Press, 1988), 277–312; Denis Wood, *The Power of Maps* (London: Routledge, 1993); and many others. In the history of science, see Simon Naylor, ‘Introduction: Historical Geographies of Science – Places, Contexts, Cartographies’, *British Journal for the History of Science*, 38, 1 (2005), 1–12, and Heather Winlow, ‘Anthropometric Cartography: Constructing Scottish Racial Identity in the Early Twentieth Century’, *Journal of Historical Geography*, 27, 4 (2001) 507–28.10.1006/jhge.2001.035418459237

5.
Camerini, *op. cit.* (note 1), 701.

6.

Veronika Lipphardt , ‘Traditions and Innovations: Visualizations of Human Variation, c. 1900–38’, History of the Human Sciences, 28, 5 (2015), 49–79, 51.

7.
For spatial imaginaries, see Josh Watkins, ‘Spatial Imaginaries Research in Geography: Synergies, Tensions, and New Directions’, *Geography Compass*, 9, 9 (2015), 508–22. See also David Livingstone, ‘Cultural Politics and the Racial Cartographics of Human Origins’, *Transactions*, 35, 2 (2010), 204–21, and David Livingstone, *Putting Science in Its Place: Geographies of Scientific Knowledge* (Chicago: University of Chicago Press, 2003).

8.
Maryinez Lyons, *The Colonial Disease: a Social History of Sleeping Sickness in Northern Zaire, 1900–40* (Cambridge: Cambridge University Press, 1992); Alison Bashford, *Imperial Hygiene: A Critical History of Colonialism, Nationalism and Public Health* (Basingstoke: Palgrave Macmillan, 2004); Warwick Anderson, *Colonial Pathologies: American Tropical Medicine, Race, and Hygiene in the Philippines* (Durham, NC: Duke University Press, 2006); Widmer and Lipphardt, *op. cit.* (note 3).

9.
For the Dutch Empire, see, for example, Hans Pols, ‘European Physicians and Botanists, Indigenous Herbal Medicine in the Dutch East Indies, and Colonial Networks of Mediation’, *East Asian Science, Technology and Society*, 3 (2009), 173–208, and Hans Pols, ‘Indo-Europeans in the Dutch East Indies: an Indo-European analysis of a paradoxical colonial category’, in Widmer and Lipphardt, *op. cit.* (note 3), 205–23.

10.
Stephanie Lawson, “‘Melanesia”, The History and Politics of an Idea’, *Journal of Pacific History*, 48, 1 (2013), 1–22. See also Nicholas Thomas, ‘The Force of Ethnology: Origins and Significance of the Melanesia/Polynesia Division’, *Anthropology Today*, 30, 1 (1989), 27–41, and Serge Tcherkézoff. ‘A Long and Unfortunate Voyage towards the “Invention” of the Melanesia/Polynesia Distinction 1595–1832’, translated from French by Isabel Ollivier, *Journal of Pacific History*, 38, 2 (2003), 175–96.

11.

John Reinold Forster , Observations Made during a Voyage round the World on Physical Geography, Natural History, and Ethic Philosophy (London: G. Robinson, 1778), 228.

12.
Jules Dumont d’Urville, *Voyage pittoresque autour du monde: Résumé général des voyages de découvertes de Magellan, Tasman, Dampier*, vol. 2 (Paris: Tenré, 1834–5), 194, cited by Chris Ballard, “‘Oceanic Negroes”: early British anthropology of Papuans, 1820–69’, in Bronwen Douglas and Chris Ballard (eds), *Foreign Bodies: Oceania and the Science of Race 1750–1940* (Canberra: ANU E Press, 2008), 157–201: 160. For the early history of racial categories in the region, see Bronwen Douglas, ‘Climate to crania: science and the racialization of human difference’, in Douglas and Ballard, *Foreign Bodies*, 33–96.

13.
John Crawfurd, *History of the Indian Archipelago*, vol. 1 (Edinburgh: Constable, 1820), 17–18; Ballard, *ibid.*, 169. Later in life, as the president of the London Ethnological Society, Crawfurd became a propagator of polygenism, the idea that different races were created separately and were not part of a single family, basing his ideas on both physical anthropology and linguistics. George Stocking, *Victorian Anthropology* (London: Collier Macmillan, 1987), 100.

14.
George Windsor Earl, ‘On the Leading Characteristics of the Papuan, Australian, and Malayo-Polynesian Nations’, *Journal of the Indian Archipelago and Eastern Asia*, 4 (1850), 66–74: 67, cited by Ballard, *op. cit.* (note 12), 174. For the remark about Flores, see George Windsor Earl, *The Native Races of the Indian Archipelago: Papuans* (London: Hippolyte Baillière, 1853), 6, and ‘Het eiland Flores’, *Tijdschrift voor Nederlandsch-Indië*, 17 (1855), 153–84: 165.

15.
Wallace first described the line in a paper presented (by Charles Darwin; Wallace was still travelling) in 1859 and published a year later: Alfred Russel Wallace, ‘On the Zoological Geography of the Malay Archipelago’, *Journal of the Proceedings of the Linnean Society: Zoology*, 4 (1860), 172–84. The map was first printed in Alfred Russel Wallace, ‘On the Physical Geography of the Malay Archipelago’, *Journal of the Royal Geographical Society of London*, 33 (1863), 217–34.

16.
Alfred Russel Wallace, ‘On the Varieties of Man in the Malay Archipelago’, *Transactions of the Ethnological Society of London*, 3 (1865), 196–215; Alfred Russel Wallace, *The Malay Archipelago* (London: Macmillan, 1869). For the influence of biogeography on British functionalism, see Henrika Kuklick, ‘Islands in the Pacific: Darwinian Biogeography and British Anthropology’, *American Ethnologist*, 23, 3 (1996), 611–38.

17.
Alfred Russel Wallace, *MS Malay Archipelago Journal*, vol. 1, 49, quoted in Jeremy Vetter, ‘Wallace’s Other Line: Human Biogeography and Field Practice in the Eastern Colonial Tropics’, *Journal of the History of Biology*, 39 (2006), 89–123: 108.

18.
Vetter, *ibid.*, 105; Ballard, *op. cit.* (note 12), 158.

19.
For Wallace’s views on colonialism, see also Mark Clement, ‘Evolution and Empire: Alfred Russel Wallace and Dutch Colonial Rule in Southeast Asia in the Mid-Nineteenth Century’, *Britain and the World*, 9, 1 (2016), 55–75, and Jeremy Vetter, ‘Politics, Paternalism, and Progressive Social Evolution: Observations on Colonial Policy in the Scientific Travels of Alfred Russel Wallace’, *Victorian Review*, 41, 2 (2015), 113–31.

20.
Ballard, *op. cit.* (note 12), note 5.

21.

P.J.B.C. Robidé van der Aa , ‘De verhouding der Papoes en Melanesiërs tot het Maleisch-Polynesische ras’, Tijdschrift van het Koninklijk Aardrijkskundig Genootschap, 2 (1885), 225–45: 227 and 243–4.

22.

Carl Meinicke , ‘Bemerkungen zu Wallace’s Ansichten über die Bevölkerung der indischen Inseln’, Zeitschrift für Ethnologie, 3 (1871), 84–93.

23.
Pieter Johannes Veth, ‘Observations sur les lignes de Wallace’, in *Congrès international des sciences géographiques tenu à Paris du 1er au 11 août 1875*, vol. 1 (Paris: Martinet, 1878), 305–9; Alfred Russel Wallace, *Insulinde: het land van den orang-oetan en den paradijsvogel. Door Alfred Russel Wallace, uit het Engelsch vertaald en van aanteekeningen voorzien door P.J. Veth* (Amsterdam: Van Kampen, 1870). For Veth, see Paul van der Velde, *A Lifelong Passion: P.J. Veth (1814–95) and the Dutch East Indies* (Leiden: KITLV Press, 2006), 181.

24.
‘Séance du 6 août 1975’, in *Congrès international des sciences géographiques tenu à Paris du 1er au 11 août 1875*, vol. 1 (Paris: Martinet, 1878), 275–80: 279.

25.

John Crawfurd , ‘On the Malayan and Polynesian Languages and Races’, Journal of the Indian Archipelago and Eastern Asia, 2 (1848), 330–74: 331.

26.
Salomon Müller, *Verhandelingen over de natuurlijke geschiedenis der Nederlandsche overzeesche bezittingen.* *Reizen en onderzoekingen 3, Land- en volkenkunde* (Leiden: S.J. Luchtmans, 1857), 252; Pierre-Adolphe Lesson, ‘Quelques mots sur les races noires de Timor’, *Revue d’Anthropologie*, 6 (1877), 256–64. See also J. Montano, ‘Sur les races des Philippines’, *Bulletins de la Société d’Anthropologie de Paris*, 7 (1884), 51–8, and N.n., ‘De verschillende menschenrassen in den Indischen Archipel’, *Tijdschrift voor Nederlandsch-Indië*, 4 (1842), 539.

27.
Ernest-Théodore Hamy, ‘Documents pour servir à l’anthropologie de l’île de Timor’, *Nouvelles archives du Muséum d’histoire naturelle de Paris*, 10 (1874), 245–68: 246; Justus Lodewijk Dusseau, *Musée Vrolik: Catalogue de la collection d’anatomie humaine, comparée et pathologique de M.M. Ger. et W. Vrolik* (Amsterdam: De Roever Kröber, 1865), 87–8.

28.
Armand de Quatrefages and Ernest- Théodore Hamy, *Crania ethnica, les cranes des races humaines décrits et figurés* (Paris: Baillière, 1882), 169–70; Ernest-Théodore Hamy, ‘Sur l’anthropologie de l’île de Timor’, *Bulletins de la Société d’Anthropologie de Paris*, 10 (1875), 224–7.

29.
George Alexander Wilken, for example, wrote in his widely read *Handleiding voor de vergelijkende volkenkunde van Nederlandsch-Indië* (Leiden: Brill, 1893) about the Malay, or Indonesian, and the Papuan, or Melanesian, race: v–xii. See also Johannes Jacobus de Hollander, *Handleiding bij de beoefening der land- en volkenkunde van Nederlandsch Oost-Indië*, 5th edition revised by R. van Eck (Breda: Broese, 1895), 111–13, and Johannes Jacobus de Hollander, ‘Een Engelschman en een Noord-Amerikaan in Nederlandsch Oost-Indië’, *Bijdragen tot de taal-, land- en volkenkunde*, 19, 1 (1872), 93–132: 106. De Hollander drew the boundary line through the island of Bacan in the Moluccas.

30.
Anthropometry was introduced as a term in the seventeenth century and initially entailed investigating the relation between body proportions and diseases and the study of the human aging process to refine the depiction of the body in art. These early measurements were taken from individuals, and only in the nineteenth century were they applied to larger groups and mobilised to answer questions about race. Frank Spencer (ed.), *History of Physical Anthropology. An Encyclopedia* (New York and London: Garland, 1997), 80–90; Gert Theile, *Anthropometrie: zur Vorgeschichte des Menschen nach Maß* (München: Fink, 2005); Christine Hanke, *Zwischen Auflösung und Fixierung: Zur Konstitution von ‘Rasse’ und ‘Geschlecht’ in der physischen Anthropologie um 1900* (Bielefeld: Transcript Verlag, 2007).

31.

Paul Topinard , ‘Observations upon the Methods and Processes of Anthropometry’, Journal of the Anthropological Institute of Great Britain and Ireland, 10 (1881), 212–24: 213.

32.
See, for example, Hanke, *op. cit.* (note 30), Tracy Teslow, *Constructing Race: The Science of Bodies and Cultures in American Anthropology* (New York: Cambridge University Press, 2014) and Fenneke Sysling, *Racial Science and Human Diversity in Colonial Indonesia* (Singapore: NUS Press, 2016).

33.
Veronika Lipphardt, “‘Geographical Distribution Patterns of Various Genes”: Genetic Studies of Human Variation after 1945’, *Studies in History and Philosophy of Biological and Biomedical Sciences*, 47 (2014), 50–61: 53; Jenny Bangham, ‘Blood Groups and Human Groups: Collecting and Calibrating Genetic Data after World War Two’, *Studies in History and Philosophy of Biological and Biomedical Sciences*, 47 (2014), 74–86.10.1016/j.shpsc.2014.05.00625049107

34.

Bruno Oetteking , ‘Lehrbuch der Anthropologie in systematischer Darstellung mit besonderer Berücksichtigung der anthropologischen Methoden, für Studierende, Arzte und Forschungsreisende by Rudolf Martin’ (review), American Anthropologist, 17, 4 (1915), 751–4: 752.

35.
Richard Neuhauss, *Deutsch Neu-Guinea*, vol. 1 (Berlin: Reimer, 1911), 93; Arnold van den Broek, ‘Über Pygmäen in Niederländisch-Süd-Neu-Guinea’, *Zeitschrift für Ethnologie*, 45 (1913), 23–44: 33; Richard Neuhauss, ‘Bemerkungen zu dem Aufsatz von A.J.P. v.d. Broek über Pygmäen in Niederländisch-Süd-Neu-Guinea’, *Zeitschrift für Ethnologie*, 45 (1913), 45–8.

36.
Elazar Barkan, *The Retreat of Scientific Racism: Changing Concepts of Race in Britain and the United States between the World Wars* (Cambridge: Cambridge University Press, 1992); Elise Smith, *Skulls, Nation and Empire: The Rise and Fall of British Craniology, 1800–1939* (Cambridge: Cambridge University Press, forthcoming).

37.
The idea that different practices sometimes produce friction is from Annemarie Mol, *The Body Multiple. Ontology in Medical Practice* (Durham, NC: Duke University Press, 2002). See also Sysling, *op. cit.* (note 32), 12–13.

38.
Helen Tilley, *Africa as a Living Laboratory: Empire, Development, and the Problem of Scientific Knowledge, 1870–1950* (Chicago: University of Chicago Press, 2011); Erik Linstrum, *Ruling Minds: Psychology in the British Empire* (Cambridge, MA: Harvard University Press, 2016).

39.
Joseph Barnard Davis, ‘On the Method of Measurements, as a Diagnostic Means of Distinguishing Human Races, Adopted by Drs Scherzer and Schwarz in the Austrian Circumnavigatory Expedition of the “Novara”’, *Transactions of the Ethnological Society of London*, 1 (1861), 123–8; Augustin Weisbach, *Reise der Österreichischen Fregatte ‘Novara’ um die Erde in den Jahren 1857, 1858, 1859* *Anthropologischer Theil 2. Körpermessungen an Individuen verschiedener Menschenracen, vorgenommen durch Karl Scherzer und Eduard Schwarz* (Vienna: government printing office, 1867).

40.
For late-nineteenth-century Dutch imperialism and the Ethical Policy, see Maarten Kuitenbrouwer, *The Netherlands and the Rise of Modern Imperialism: Colonies and Foreign Policy, 1870–1902* (New York: Berg, 1991), Elsbeth Locher-Scholten, *Ethiek in fragmenten. Vijf studies over koloniaal denken en doen van Nederlanders in de Indonesische Archipel, 1877–1942* (Utrecht: Hes, 1981) and Elsbeth Locher-Scholten, ‘Dutch Expansion in the Indonesian Archipelago around 1900 and the Imperialism Debate’, *Journal of Southeast Asian Studies*, 25 (1994), 91–111.

41.

Lindor Serrurier , De Pionier: Handleiding voor het verzamelen en waarnemen op natuurkundig gebied, inzonderheid in tropische gewesten (Leiden: Brill, 1891), 135–136.

42.
Pieter Hovens, *Herman F.C. ten Kate Jr. (1858–1931) en de antropologie der Noord-Amerikaanse Indianen* (Meppel: Krips Repro, 1989), 40; Herman ten Kate, ‘Over antropologisch onderzoek op reis’, *Tijdschrift van het Koninklijk Nederlandsch Aardrijkskundig Genootschap*, 6 (1882), 254–8; Herman ten Kate, ‘Beschouwingen over eenige anthropologische vraagstukken’, *Tijdschrift van het Koninklijk Nederlandsch Aardrijkskundig Genootschap*, 30 (1913), 719–41.

43.
Herman ten Kate, ‘Contribution à l’anthropologie de quelques peuples d’Océanie’, *L’Anthropologie*, 4 (1893), 279–300: 280; Herman ten Kate, ‘Verslag eener reis in de Timoergroep en Polynesië’, *Tijdschrift van het Koninklijk Nederlandsch Aardrijkskundig Genootschap*, 11 (1894), 195–246, 333–90, 541–636, 659–700: 340–1.

44.
Ten Kate, ‘Verslag eener reis’, *ibid.*, 588.

45.
Ten Kate, ‘Contribution à l’anthropologie’, *op. cit.* (note 43), 289.

46.
In Europe, things were different again, with Germanic people generally seen as long-headed, and Alpine and Celtic people seen as short-headed. Broad skulls were also linked to people living at higher altitudes. See ten Kate, ‘Beschouwingen over eenige anthropologische vraagstukken’, *op. cit.* (note 42).

47.
Ten Kate, ‘Contribution à l’anthropologie’, *op. cit.* (note 43), 290, 294.

48.
Ten Kate, ‘Verslag eener reis’, *op. cit.* (note 43), 220, 342, 553, 557, 689; Herman ten Kate, ‘Mélanges anthropologiques III. Indigènes de l’Archipel Timorien’, *L’Anthropologie*, 26 (1915), 519–54: 519.

49.

Herman Ten Kate , ‘Résumé des principaux résultats scientifiques du voyage, Tijdschrift van het Koninklijk Nederlands Aardrijkskundig Genootschap, 2 (1894), 819–822.

50.

Hendrik Bijlmer , Outlines of the Anthropology of the Timor Archipelago (Weltevreden: Kolff, 1929), 7.

51.

Hendrik Bijlmer , ‘Een anthropologische exploratietocht naar Insulinde’s Zuidoosthoek, Mensch en Maatschappij, 6 (1930), 99–113: 100.

52.

*Ibid.*, 99.

53.
Bijlmer, *op. cit.* (note 50), 91.

54.
Bijlmer, *op. cit.* (note 50), 97. At about the same time, J.A. van Staveren published an article in which he stated, like Bijlmer, that the Papuan types increased on Flores from west to east: J.A. van Staveren, ‘De Rokka’s van Midden-Flores’, *Tijdschrift voor Indische Taal-, Land- en Volkenkunde*, 57 (1915), 117–75.

55.
Bijlmer, *op. cit.* (note 50), 36, 189–90.

56.

Doeke Brouwer , Bijdrage tot de anthropologie der Aloreilanden (Amsterdam: Uitgeversmaatschappij Holland, 1935).

57.
Wilhelmina Keers, *An Anthropological Survey of the Eastern Little Sunda Islands* (Amsterdam: Indisch Instituut, 1948). See also Wilhelmina Keers, ‘Colour of the Eyes, Hair and Skin of the Population of the Manggarai’, *Acta Neerlandica Morphologiae Normalis et Pathologicae*, 3 (1939), 35–43. Adèle van Bork-Feltkamp, another female anthropologist, never visited the Timor archipelago but wrote *A Contribution to the Anthropology of Timor and Roti after Data Collected by Dr W.L. Meyer* (Amsterdam: KIT, 1951). She emphasised the usefulness of a comparison between a group of Timorese living in the mountains and the highland Papuans of New Guinea. See also J.A.J. Barge, ‘Contributions to the Anthropology of Timor and Flores (Dutch East Indies) after Data Collected by Dr B. Vroklage s.v.d.’, *Acta Neerlandica Morphologiae Normalis et Pathologicae,* 4 (1941–2), 329–56, and 5 (1943–5), 119–30, 213–28, 262–73.

58.
Sysling, *Racial Science and Human Diversity* For anthropology and imperialism, see, among many others, Jan van Bremen and Akitoshi Shimizu (eds), *Anthropology and Colonialism in Asia and Oceania* (Richmond: Curzon, 1999), Helen Tilley and Robert Gordon (eds), *Ordering Africa: Anthropology, European Imperialism and the Politics of Knowledge* (Manchester: Manchester University Press, 2007) and Emmanuelle Sibeud, ‘A Useless Colonial Science? Practicing Anthropology in the French Colonial Empire, circa 1880–1960’, *Current Anthropology*, 53, suppl. 5 (2012), 83–94.

59.
Ricardo Roque, ‘The Colonial Ethnological Line: Timor and the Racial Geography of the Malay Archipelago’, *Journal of Southeast Asian Studies*, 49 (2018), 387–409. See also António Mendes Correia, *Antropologia Timorense* (Porto: Tipografia da Renascença Portuguesa, 1916) and António Mendes Correia, *Timor Português. Contribuições para o seu Estudo Antropológico* (Lisbon: Ministério das Colónias, 1944). In the 1940s, Huub Lammers again confirmed that (pre-) Malay influence decreased from east to west: Huub Lammers, *De physische anthropologie van de bevolking van Oost-Dawan (Noord-midden-Timor)* (Leiden: s.n., 1948), 273.

60.
Arend Lijphart, ‘The Indonesian Image of West Irian’, *Asian Survey*, 1, 5 (1961), 9–16: 11. See also R.E. Elson, *The Idea of Indonesia* (Cambridge: Cambridge University Press, 2008), 34, 63.

61.
Deliar Noer, ‘Yamin and Hamka: two routes to an Indonesian identity’, in A. Reid and David Marr (eds), *Perceptions of the Past in Southeast Asia* (Singapore: Heinemann, 1979), 249–62: 257; Adrian Vickers, *A History of Modern Indonesia* (Cambridge: Cambridge University Press, 2005), 139.

62.

*Rapport van de commissie Nieuw-Guinea (Irian) 2. Tekst en bijlagen* (The Hague: Secretariaat van de Nederlands-Indonesische Unie, 1950), 18.

63.

Vincent Kuitenbrouwer , ‘Beyond the “Trauma of Decolonisation”: Dutch Cultural Diplomacy during the West New Guinea Question (1950–62), Journal of Imperial and Commonwealth History, 44, 2 (2016), 306–323.

64.

David Webster , ‘Race, identity and diplomacy in the Papua decolonization struggle, 1949–62, in Philip Muehlenbeck (ed.), Race, Ethnicity and the Cold War: A Global Perspective (Nashville, TN: Vanderbilt University Press, 2012), 91–117: 91–2.

65.

Johan Ariks , De Papoea’s roepen Nederland: een dringend beroep van de Papoea-delegatie op het Nederlandse volk (Amsterdam: C. de Boer, 1951), 2.

66.
Markus Kaisiepo and Nicolaas Jouwe, *The Voice of the Negroids of the Pacific to the Negroids throughout the World* (The Hague: A.W. Dijkman, 1962), 6. Kaisiepo was sometimes happy to include the Moluccas in the Papuan/Melanesian sphere: ‘Geen actie van de Papoea’s tegen bezoek Soeharto’, *Nieuwe Leidsche Courant*, 12 (September 1970), 9, 26.

67.

*The Question of West Irian in the United Nations, 1954–7* (Jakarta: Ministry of Foreign Affairs Indonesia, 1958), 25; see also 37–9.

68.
Webster, *op. cit.* (note 64), 97.

69.

A. Ananta , ‘Statistics on Ethnic Diversity in the Land of Papua, Indonesia, Asia and the Pacific Policy Studies, 3, 3 (2016), 458–474.

70.
Ministry of Information, *The Truth about West Irian* (1956), 5–7, cited by Webster, *op. cit.* (note 64), 91–117: 96.

71.

Compare Lawson , *op. cit.* (note 10).

